# Corrigendum to “Design of a phase 3, randomized, double-blind, placebo-controlled study to evaluate the efficacy and safety of prucalopride in pediatric patients with functional constipation” [Contemp. Clin. Trials Commun. 33 (2023) 101144]

**DOI:** 10.1016/j.conctc.2024.101302

**Published:** 2024-04-27

**Authors:** Carmen Cuffari, William Spalding, Heinrich Achenbach, Manoj Thakur, André Gabriel

**Affiliations:** a Children's National Hospital, Division of Gastroenterology, Hepatology and Nutrition, Washington, DC, USA; b Takeda Development Center Americas, Inc., Lexington, MA, USA; c Takeda Development Center Americas, Inc., Zurich, Switzerland

The authors regret that the following text was not included in the original publication for Fig. 1 to reflect that permissions have been obtained for reuse of this image: “Figure reproduced from: Design of a phase 3, randomized, double-blind, placebo-controlled study to evaluate the efficacy and safety of prucalopride in pediatric patients with functional constipation. Cuffari C et al. Copyright © 2021 ESPGHAN and NASPGHAN. Reproduced with permission of John Wiley & Sons Inc."

The authors would like to apologise for any inconvenience caused.

